# 167. Clinical Impact of Rapid Multiplex Polymerase Chain Reaction-Based Diagnostic Testing for Hospitalized Patients with Bloodstream Infections (BSIs) in a National Cohort of Veterans Affairs Patients

**DOI:** 10.1093/ofid/ofac492.245

**Published:** 2022-12-15

**Authors:** Nicholas Britt, Karim Khader, Tao He, Tristan T Timbrook, Tina M Willson, Atim Effiong, Thomas Lodise

**Affiliations:** University of Kansas School of Pharmacy, Kansas City, Kansas; University of Utah, Salt Lake City, Utah; University of Utah, Salt Lake City, Utah; bioMerieux, Salt Lake City, Utah; University of Utah, Salt Lake City, Utah; University of Utah, Salt Lake City, Utah; Albany College of Pharmacy and Health Sciences, Albany, New York

## Abstract

**Background:**

Polymerase chain reaction (PCR)-based diagnostic testing for BSIs, such as the BioFire FilmArray® Blood Culture Identification (BCID) Panel, has the potential to shorten time to appropriate therapy and duration of overly broad-spectrum antibiotics. The clinical benefit of PCR-based diagnostic technologies for BSIs has been demonstrated but data supporting their utility in larger multi-center settings are limited. This study sought to evaluate outcomes associated with the implementation of the BioFire BCID in the US Veterans Affairs (VA) system.

**Table:**
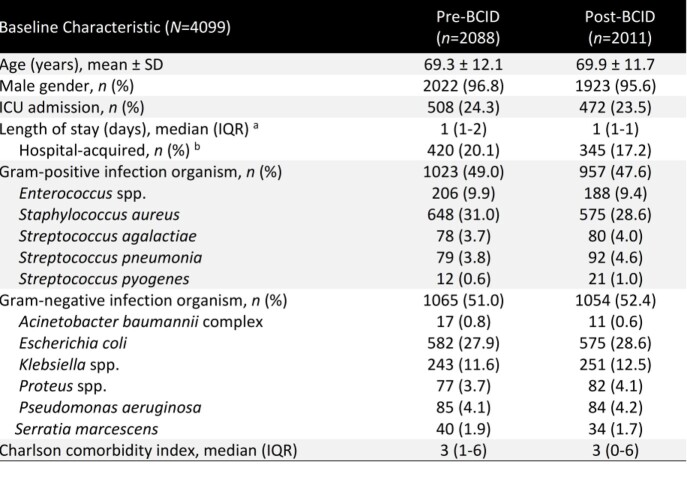

**Methods:**

A retrospective multi-center pre-post quasi-experimental study was performed among patients admitted to any VA hospital that adopted BioFire BCID Panel for ≥ 1 year. Patients with ≥ 1 BSI due to bacteria on the BioFire BCID Panel (19 bacterial targets) were included. Exclusion criteria were polymicrobial BSI or death within 48 hours of BSI. Patients were categorized into two groups: i) BSI in the 1-year prior to implementation (pre-BCID) and ii) 1-year post implementation (post-BCID). Post-BCID period commenced 2 months after first BCID-BSI. Outcomes included early de-escalation, defined as a reduction in antimicrobial spectrum score within 48 hours of index BSI, time to appropriate therapy, 30-day mortality, and 90-day CDI incidence.

**Results:**

A total of 4099 patients across 33 VA facilities met study criteria (*n*=2088 pre-BCID; *n*=2011 post- BCID). Groups were similar at baseline (**Table**). BioFire BCID implementation was associated with an improvement in early de-escalation (38.3% in post-BCID vs 34.8% in pre-BCID, p=0.02) and reduction in median (IQR) time to appropriate therapy (8 [4-19] hours in post-BCID vs 9 [3-19] hours in pre-BCID, P< 0.01). There was no significant difference in 30-day mortality (11.1% in post-BCID vs 12.4% in pre-BCID, p=0.2) or CDI (4.6% in post-BCID vs 4.8% in pre-BCID, p=0.8) between BCID groups.

**Conclusion:**

In this large VA cohort study, implementation of the BioFire BCID panel was associated with a significant improvement in early antimicrobial de-escalation and time to appropriate therapy. These data suggest this rapid diagnostic technology aids in optimization of antimicrobial use in hospitalized VA patients with BSIs.

**Disclosures:**

**Nicholas Britt, PharmD, MS**, Gilead Sciences: Advisor/Consultant|Gilead Sciences: Grant/Research Support|Merck: Advisor/Consultant|Merck: Grant/Research Support|Shionogi: Grant/Research Support **Karim Khader, PhD**, BioFire Diagnostics: Grant/Research Support **Tristan T. Timbrook, PharmD**, bioMerieux: Employee of bioMerieux **Thomas Lodise, Jr., Pharm.D., PhD**, BioFire Diagnostics: Grant/Research Support|cidara: Advisor/Consultant|cidara: Honoraria|Entasis: Grant/Research Support|Merck: Advisor/Consultant|Merck: Grant/Research Support|Paratek: Advisor/Consultant|Shionogi: Advisor/Consultant|Spero: Advisor/Consultant|Venatrox: Advisor/Consultant.

